# Letter from Dr. T. B. Gunning

**Published:** 1875-05

**Authors:** Thomas Brian Gunning

**Affiliations:** Missouri Dental Journal


### ARTICLE III.

### *Letter from Dr. Gunning.*

The following is the closing portion of a letter dated Nov. 27th, 1874, to the Dean of the Faculty of the Maryland Dental College :

"I allowed my name to be enrolled as Regent in the belief that your college was organized to advance the true interests of the profession, and, as inseparably connected therewith, the welfare of the public, and I accepted the honor of presiding at the Board in the trust that with five Regents residing in the immediate neighborhood, fitted so eminently to a wise guidance of administration by their character, experience and culture, such counsels would prevail with the Faculty that its course would be steadily forward. I will not conceal my disappointment that this hope does not appear to be realized, and I ought not to occupy a position which may be misunderstood. I must, therefore, resign, with regret, all official connection with the Maryland Dental College."

Very respectfully yours, &c.,

*Missouri Dental Journal.* THOMAS BRIAN GUNNING.

---

In answer to several letters concerning the above and the "Protest" published in pamphlet form by Dr. Gunning, we desire to inform our correspondents that the Institution referred to is *not* the old "Baltimore College," but a new one started a year or two ago; and that the only injury resulting to the "Baltimore College" has been in the mistakes of those who are led to believe the institution referred to is the old Baltimore College. We do not desire the "Baltimore College" to be confounded with the one in question.

